# The importance of tissue confirmation of metastatic disease in patients with breast cancer: lesson from a brain metastasis case

**DOI:** 10.18632/oncoscience.320

**Published:** 2016-09-12

**Authors:** Jingxian Ding, Pinghua Hu, Jun Chen, Xiaobo Wu, Yali Cao

**Affiliations:** ^1^ Department of Radiotherapy, Breast Cancer Institute, The Third Hospital of Nanchang, Nanchang 330009, China; ^2^ Department of Breast Surgery, Breast Cancer Institute, The Third Hospital of Nanchang, Nanchang 330009, China

**Keywords:** breast cancer, neoadjuvant chemotherapy, ER/PR, HER2, metastatic lesion

## Abstract

**Background:**

The discrepancy of estrogen receptor (ER), progesterone receptor (PR), and human epidermal growth factor receptor 2 (HER2) statuses in breast cancers has been reported. Available systemic therapy for patients with breast cancer is based on the molecular subtypes as identified by IHC and/or FISH. However, these biomarkers may change throughout tumor progression.

**Case presentation:**

We report a relatively uncommon case of a 39-year-old Chinese woman with local advanced breast cancer (LABC) treated with 6 cycles of docetaxel, doxorubicin and cyclophosphamide (TAC) regimen neoadjuvant chemotherapy, and subsequently mastectomy, intensity-modulated radiation therapy (IMRT) and tamoxifen followed as regularly. Brain metastatic event appeared in 6 months after mastectomy. Treatment for brain metastasis was surgical resection and followed by whole brain radiotherapy (WBRT) approved by multidisciplinary team (MDT). Initial pathological diagnosis was IDC, cT4N1M0, luminal B (ER+ 90%, PR+90%, HER2 0, Ki67+ 70%) based on ultrasound-guided core needle biopsy. Surgical pathology revealed IDC, pT2N3M0 luminal B (ER+ 20%, PR+20%, HER2 0, Ki67+ 20%). Histological response to neoadjuvant chemotherapy is grade 3 according to the Miller/Payne grading system. Final pathology of brain metastasis showed a HER2 overexpression metastatic breast cancer luminal B (ER+ 70%, PR+ 70%, HER2 2+, Ki67+ 30%), FISH confirmed HER2 overexpression. Weekly paclitaxel plus trastuzumab was given for 12 weeks, then trastuzumab every 3 weeks for a whole year. Patient follow-up is still ongoing, no new events appear yet.

**Conclusions:**

The determination of hormone receptors and HER2 status should be routinely performed in all involved tissues, if possible, and systemic therapy should be tailored following the latest finding.

## INTRODUCTION

Breast cancer is one of the most common malignancies in women, and its incidence has continuously increased in recent years [1]. Locally advanced breast cancer (LABC) accounts for about 15% of newly diagnosed cases in our center, most of who come from rural countryside for lack of attention. Neoadjuvant chemotherapy was usually given to these patients in an attempt to downstage the primary tumor and also to reduce or eliminate micrometastatic disease [2, 3]. Available systemic therapies for breast cancer patients are based on the estrogen receptor (ER) and progesterone receptor (PR) and human epidermal growth factor receptor 2 (HER2) characteristics as identified by IHC and/or FISH in the tissue acquired by ultrasound-guided core needle biopsy [4–6]. In routine clinical practice, management of patients with metastatic breast cancer is also referred to the biological traits of the primary tumor. However, hormone receptors and HER2 status may change during tumor progression from the primary tumor to the metastatic side. Accumulating studies have indicated that there may be of clinical significance in discrepancy of ER, PR, and HER2 status between primary breast tumor and metastatic disease [5, 7–11]. Normally, this phenotype discordance suggests an even worse prognosis. Consequently, biopsies of metastatic tissue should be taken into account as a routine procedure in daily clinic, and these biomarkers confirmation at recurrence or metastatic carcinomas may potentially get clinically significant benefits to improve patient management and survival.

Here, we presents a relatively uncommon case with a HER2 negative breast cancer switching into HER2 overexpression breast cancer after a series of systemic therapies.

## CASE PRESENTATION

**Figure 1:**
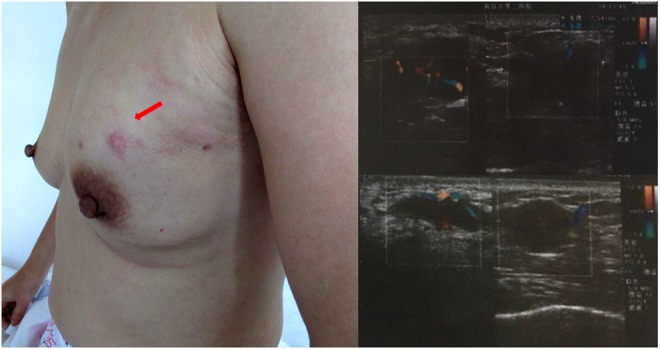
Initial clinical manifestation of the patient A red nodule located in the left upper side of left breast and several palpable lymph nodes in the homolateral axillary fossa region. Diagnostic ultrasound demonstrates hypoechoic mass and suspicious left axillary lymph nodes at initial presentation (pre-biopsy).

**Figure 2:**
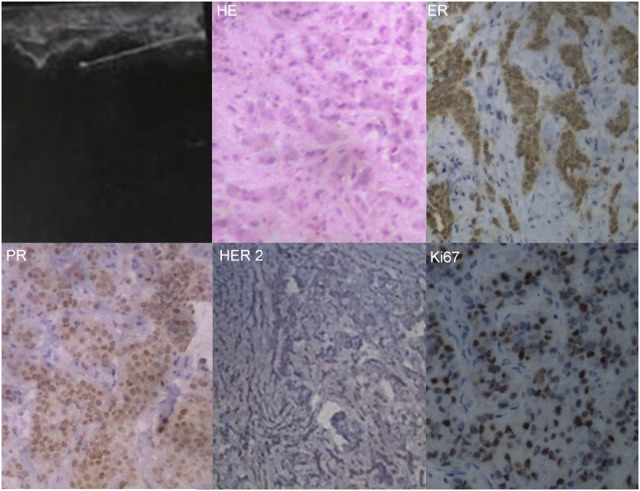
Tissue pathological confirmation of the primary mass Ultrasound-guided core needle biopsy, hematoxylin-eosin (HE) morphological diagnosis and immunohistochemistry examination routinely.

A 39-year-old Chinese woman with local advanced breast cancer (LABC) as pathologically confirmed by core needle biopsy in our breast cancer center. Before coming to my clinic, she was treated with Traditional Chinese Medicine for misdiagnosis as breast hyperplasia in local hospital for about one year, no obvious symptom improvement as she mentioned. A red nodule appeared in the left upper side of left breast one month before she came to my clinic (Figure 1), which made her come to our breast cancer center. Color Doppler Ultrosonography for the left breast demonstrated a left-sided hypoechoic mass measuring 3.5 cm and located at the 3 o'clock position adjacent to the nipple-areolar complex, and also revealed suspicious left axillary lymph nodes (Figure 1). Ultrasound-guided biopsy of the breast mass demonstrated an infiltrating ductal carcinoma (IDC) of the left breast with ER+ 90% mild, PR+90% mild, HER2 0, Ki67+ 70% by immunohistochemistry (IHC), luminal B subtype (Figure 2). No detectable involved organs as screened by systemic assessment, including brain, lungs, liver, bone, and uterus and its accessories. The clinical stage of the case was cT4N1M0 based on American Joint Committee on Cancer Breast Cancer Staging 7th edition [12].

**Figure 3:**
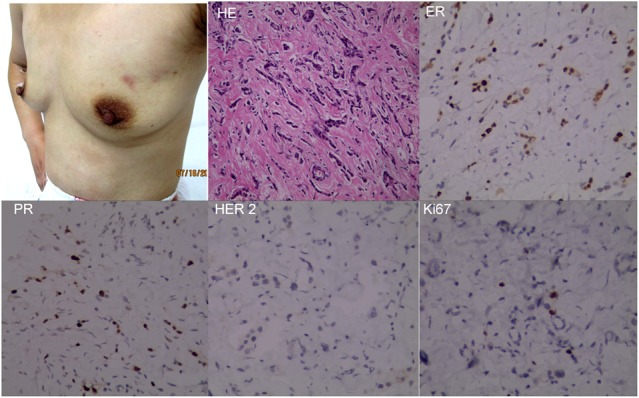
Reassessment of the primary tumor after neoadjuvant chemotherapy Skin red nodule disappeared after 6 cycles of TAC neoadjuvant chemotherapy. Surgical pathology revealed a similar subtype of breast cancer with core needle biopsy before neoadjuvant chemotherapy, though ER, PR and Ki67 staining intensity was not completely consistent, which didn't change the subtype of breast cancer.

**Figure 4:**
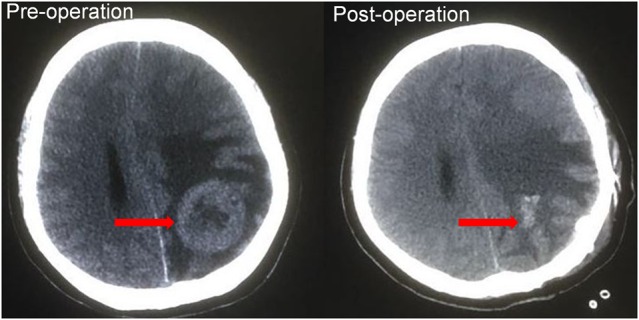
Cranial computerized tomography (CT) CT revealed the brain metastatic lesion and brain midline shift. There is a slightly high density nodule in the left parietal lobe, and the surrounding is the low density edema before operation, and patchy opacity left there after operation.

**Figure 5:**
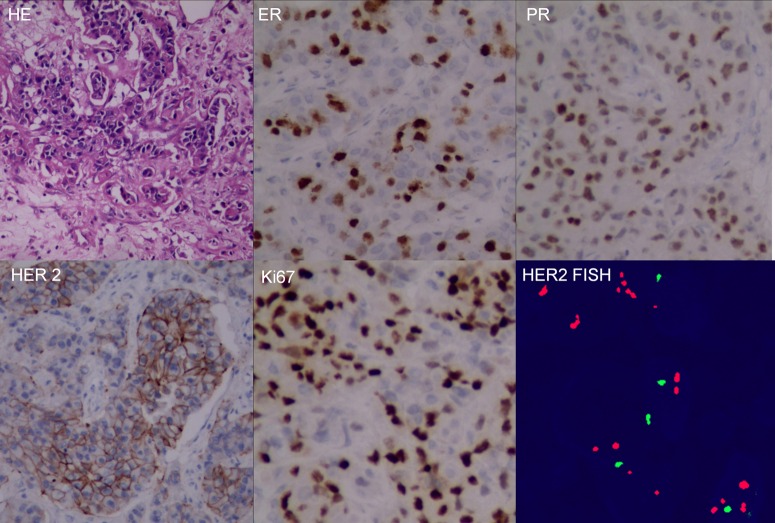
Surgical pathology of the brain metastatic lesion Hematoxylin-eosin (HE) morphologically identified breast cancer metastasis in brain, and immunohistochemistry examination reassessment of the metastatic lesion showed ER+70% mild, PR+70% mild, HER2++, Ki67+ 30%, fluorescence in situ hybridization confirmed HER2 overexpression.

The patient received 6 cycles of docetaxel, doxorubicin and cyclophosphamide (TAC) regimen neoadjuvant chemotherapy. Clinical assessment is partial response (PR) after 6 cycles of neoadjuvant chemotherapy, and then mastectomy. Surgical pathology revealed IDC, pT2N3M0, luminal B (ER+ 20% weak, PR+20% weak, HER2 0, Ki67+ 20%). Histological response to neoadjuvant chemotherapy is grade 3 according to the Miller/Payne grading system [13] (Figure 3). Forward planning intensity-modulated radiation therapy (IMRT) and then tamoxifen followed. The target volume of radiotherapy included the chest-wall and supraclavicular lymphonodus drawing region. Brain metastatic event appeared in 6 months after mastectomy as firstly presented by terrible headache and intracranial hypertension, which were confirmed by cranial computerized tomography (Figure 4). Emergency management of brain metastasis was surgical resection and followed by whole brain radiotherapy (WBRT) approved by multi-disciplinary team (MDT). Final pathology of brain metastasis showed a HER2 overexpression metastatic breast cancer with ER+ 70% mild, PR+ 70% mild, HER2 2+, Ki67+ 30%), FISH confirmed HER2 overexpression (Figure 5). Weekly paclitaxel plus trastuzumab was given to this patient for 12 weeks, then trastuzumab every 3 weeks for a whole year. Endocrine therapy switched into ovarian function suppression plus exemestane according to the latest clinical evidence [14]. Patient follow-up is still ongoing, the last follow-up is February 20th 2016, and no new events appear yet.

## DISCUSSION

Breast cancer threatens womens’ health worldwide [15]. It is the second most common malignancy in China [16, 17]. Breast cancer includes hormone-dependent and hormone-independent tumor based on the expression of ER and/or PR status, which is the important indicator for efficacy and prognosis in patients with endocrine therapies [6]. According to current National Comprehensive Cancer Network (NCCN) guidelines, endocrine treatment is indicated in all patients with a positive hormone receptor (HR) status, which is defined as ER positive and/or PR positive. The human epidermal growth factor receptor 2 (HER2) is amplified in approximately 15-25% of breast cancers. HER2 overexpression in breast cancer has been associated with tumor invasiveness, progressive regional and distant metastases, and poor prognosis [18–21]. The NCCN guideline recommends molecularly targeted therapy for first-line treatment of patients with HER2- positive metastatic breast cancer. Neoadjuvant chemotherapy is gradually used for LABC and there is a trend for tailored therapies based on molecular subtypes of breast cancer. The accuracy of the core needle biopsy (CNB) for determination of the hormone receptor status in breast cancer patients has been extensively studied and can be used with confidence for ER and HER2 determination. However, the results for PR are more variable and need to be used with caution [22]. Neoadjuvant therapy is mainly based on the immunohistochemical findings of the HR and HER2 status on the core needle biopsy. With the growing use of neoadjuvant therapy, it is important to know whether it modulates the biological behaviors of breast cancer cells [3, 23, 24].

However, we know little about the impact of neoadjuvant chemotherapy drugs on those biomarkers and the possible consequences for subsequent systemic salvage therapy at present time. Nowadays, the evaluation of ER, PR and HER-2 status is mainly through IHC or FISH. The assessment was generally performed on the primary neoplasm in the assumption that the status should remain stable in most of the cases as demonstrated in previous reports. Moreover, in certain circumstance, the biopsy of metastatic site is not an easy task, such as brain metastasis and metastasis in deep organs. HER2 overexpression is recognized to be of strong predictive value in the treatment with HER2 inhibitors. It is reported that patients with HER2 positive breast cancer have better responses and higher pCR rates when adds trastuzumab to neoadjuvant chemotherapy [25–27].

In fact, some reports have suggested that ER, PR, and HER2 status switch between primary breast cancer and metastatic sites, and therefore, the confirmation of hormone receptors and HER2 status of metastatic sites should be routinely performed, which together with that of primary tumor to provide evidence for the choice of systemic salvage therapies. Several studies have carried out retrospective analyses comparing the ER, PR, and HER2 status of primary tumors and paired metastasis. For example, Fabris et al identified HER-2 status on 119 cases of primary infiltrating breast carcinoma and paired metastases. Therapeutically significant HER-2 status discordance was verified between primary carcinoma and synchronous lymph node metastases (6.7%), local recurrence (13.3%) and metachronous distant metastases (28.6%). In the comparison, they found that both normal HER-2 status in primary tumors to HER-2 amplification in paired metastases and HER-2 overexpression in primary tumors to normal HER-2 status in metastatic sites were evident. Taking together, 14 out of 65 cases (21.5%) showed a therapeutically significant discordance of HER-2 status between the primary tumor and the paired metachronous recurrence or metastasis, the 15.4% of cases showing normal HER-2 status in the primary tumor and HER-2 overexpression in the metastatic sites [28]. A convincing explanation for this phenomenon is still unborn. Nevertheless, controversial opinions do exist both about the stability of HER-2 status in breast carcinoma throughout the course of the disease, and about whether chemotherapy (neoadjuvant or adjuvant) may modify HER2 expression. Such as a possible genetic drift or clonal selection for HER-2 which may happen during tumor progression, intratumoral heterogeneity of HER-2 status, or a clone selection of having enhanced metastatic potential. The authors demonstrated that these marker reinvestigations at metastatic sites may potentially improve patient management and survival [29–31].

In our daily clinic, the discordance of the biomarkers is not uncommon. Nevertheless, little attention was paid. The case presented here sound the alarm for us to emphasize importance of tissue confirmation of metastatic disease in patients with breast cancer.

## CONCLUSION

A change in hormone receptor or HER2 status would have important therapeutic, prognostic and financial consequences for both patients and health care providers. Though data on the influence of neoadjuvant therapy on the expression status of ER, PR and HER2 are few, even some reports show controversial results. We strongly recommend that ER, PR and HER2 of recurrent or metastatic lesions should usually be confirmed whenever possible, especially for patients whose clinical manifestations are different from the biomarker characteristics of the primary tumors. Such as a short natural history of the disease, site(s) of recurrence, co-morbidities and previous treatments. Subsequent treatment measures should be modulated accordingly. Moreover, this procedure may be also recommended in the patients who are metastatic at the time of diagnosis.
